# The synthesis and characterization of monodispersed chitosan-coated Fe_3_O_4_ nanoparticles via a facile one-step solvothermal process for adsorption of bovine serum albumin

**DOI:** 10.1186/1556-276X-9-296

**Published:** 2014-06-11

**Authors:** Mao Shen, Yujing Yu, Guodong Fan, Guang Chen, Ying min Jin, Wenyuan Tang, Wenping Jia

**Affiliations:** 1 College of Pharmaceutical and Chemical Engineering, Taizhou University, Jiaojiang 318000, People's Republic of China; 2 Key Laboratory of Auxiliary Chemistry and Technology for Chemical Industry, Ministry of Education, Shan xi University of Science and Technology, Xi'an 710021, People's Republic of China; 3 College of Pharmaceutical Sciences, Zhejiang University, Hangzhou 310058, People's Republic of China

**Keywords:** Magnetic nanoparticles, Chitosan, Solvothermal, BSA adsorption

## Abstract

Preparation of magnetic nanoparticles coated with chitosan (CS-coated Fe_3_O_4_ NPs) in one step by the solvothermal method in the presence of different amounts of added chitosan is reported here. The magnetic property of the obtained magnetic composite nanoparticles was confirmed by X-ray diffraction (XRD) and magnetic measurements (VSM). Scanning electron microscopy (SEM) and transmission electron microscopy (TEM) allowed the identification of spherical nanoparticles with about 150 nm in average diameter. Characterization of the products by Fourier transform infrared spectroscopy (FTIR) demonstrated that CS-coated Fe_3_O_4_ NPs were obtained. Chitosan content in the obtained nanocomposites was estimated by thermogravimetric analysis (TGA). The adsorption properties of the CS-coated Fe_3_O_4_ NPs for bovine serum albumin (BSA) were investigated under different concentrations of BSA. Compared with naked Fe_3_O_4_ nanoparticles, the CS-coated Fe_3_O_4_ NPs showed a higher BSA adsorption capacity (96.5 mg/g) and a fast adsorption rate (45 min) in aqueous solutions. This work demonstrates that the prepared magnetic nanoparticles have promising applications in enzyme and protein immobilization.

## Background

In the past several decades, magnetic nanomaterials of iron oxides (Fe_3_O_4_ NPs) have attracted much research interest due to their potential applications in magnetic storage, catalysis, electrochemistry, drug delivery, medical diagnostics, and therapeutics based on their unique magnetic, physiochemical, and optical properties [1-5]. Among the various methods for the preparation of Fe_3_O_4_ NPs, the solvothermal approach is one of great significance [6-9]. Under the solvothermal conditions, Fe_3_O_4_ NPs were usually composed of multiple single-domain magnetic nanocrystals. To date, the solvothermal method was developed for the preparation of magnetite spheres with strong magnetization through the hydrolysis and reduction of iron chloride in ethylene glycol at high temperatures. However, producing Fe_3_O_4_ NPs with specific functional groups on the surface and acceptable size distribution without particle aggregation has consistently been a problem. Thus, a variety of modifiers were added to the reaction mixtures to control the size of Fe_3_O_4_ NPs and improve the colloidal stability and biocompatibility, such as poly(acrylic acid) (PAA) [10], polyethyleneimine (PEI) [11,12], polyethylene glycol (PEG) [13], and other biocompatible polymers [14,15]. These modifiers are usually polymers bearing carboxylate or other charged groups. During the formation process of Fe_3_O_4_ NPs, these charged groups can coordinate with iron cations in solution, and affect the nucleation and aggregation of the nanocrystals, resulting in the formation of Fe_3_O_4_ NPs with controllable grain size and self-assembled structures. Compared with the types of polymers mentioned above, chitosan has been intensively studied as a base material for magnetic carriers because of its significant biological and chemical properties. The conventional method for preparing Fe_3_O_4_ NPs coated with chitosan is the coprecipitation method that involves obtaining the magnetic nanoparticles, followed by chitosan coating. Several research teams have tried to simplify the procedure to obtain Fe_3_O_4_ NPs coated with chitosan in one step [16-20]. However, there are very few reports on the synthesis of magnetic nanoparticles coated with chitosan (CS-coated Fe_3_O_4_ NPs) by a one-step solvothermal process.

In this paper, we report the preparation of monodispersed CS-coated Fe_3_O_4_ NPs in the presence of different amounts of added chitosan via a facile one-step solvothermal process. A detailed characterization of the products was carried out to demonstrate the feasibility of this method for obtaining CS-coated Fe_3_O_4_ NPs. Bovine serum albumin (BSA) isolation experiments were used to demonstrate the potential of the materials for adsorption.

## Methods

### Chemicals

Ferric chloride hexahydrate (FeCl_3_ · 6H_2_O, >99%), anhydrous sodium acetate (NaOAc), ethylene glycol (EG), polyvinylpyrrolidone (PVP), bovine serum albumin (BSA), and chitosan (low molecular weight, Brookfield viscosity 20 cps) were purchased from Aldrich (St. Louis, MO, USA). The pure water was obtained from a Milli-Q synthesis system (Millipore, Billerica, MA, USA).

### Preparation of CS-coated Fe_3_O_4_ NPs

Functionalized magnetite nanoparticles were synthesized via a versatile solvothermal reaction reported by Li with a slight modification [21]. Typically, FeCl_3_ · 6H_2_O (1.50 g), chitosan (with various chitosan/Fe weight ratios: 0, 1/3, 1/2, 2/3, 5/6, 1), NaOAc (3.6 g), and PVP (1.0 g) were added to 70 mL of ethylene glycol to give a transparent solution via vigorous stirring. This mixture was then transferred to a Teflon-lined autoclave (80 mL) for treatment at 200°C for 8 h. The composite nanoparticles were denoted MFCS-0 (naked Fe_3_O_4_), MFCS-1/3, MFCS-1/2, MFCS-2/3, MFCS-5/6, and MFCS-1. The products were obtained with the help of a magnet and washed with 0.5% dilute acetic acid and demonized water. Finally, the products were collected with a magnet and dried in a vacuum oven at 60°C for further use.

### Characterization

Transmission electron microscopy (TEM) images were obtained with a JEM-2100 transmission electron microscope (Jeol Ltd., Tokyo, Japan). X-ray diffraction (XRD) analysis was performed using a Dmax-2500 (Rigaku Corporation, Tokyo, Japan). Magnetic measurements (VSM) were studied using a vibrating sample magnetometer (Lake Shore Company, Westerville, OH, USA) at room temperature. Scanning electron microscopy (SEM) images were carried out on a Philips XL30 microscope (Amsterdam, The Netherlands). The zeta potential of these particles was measured by dynamic light scattering (DLS) with a Delsa™ NanoC Particle Size Analyzer (Beckman Coulter, Fullerton, CA, USA). Thermogravimetric analysis (TGA) of the nanocomposite and chitosan was performed in a TGA Q500 from TA Instruments (New Castle, DE, USA). Analyzed samples were heated from 100°C to 800°C at a heating rate of 10°C/min under a nitrogen flow of 50 mL/min. Fourier transform infrared spectroscopy (FTIR) of the nanocomposite and chitosan was performed by Nicolet 5700 (Thermo Nicolet, Waltham, MA, USA). The adsorption of BSA on CS-coated Fe_3_O_4_ NPs was measured using a UV-2501PC spectrometer (Shimadzu Corporation, Tokyo, Japan).

### Adsorption procedures of BSA

Adsorption of BSA on the CS-coated Fe_3_O_4_ NPs was carried out by mixing 10 mg of dried CS-coated Fe_3_O_4_ NPs and 10 mL of BSA solution (0.1, 0.2, 0.3, and 0.4 mg/L, pH = 6.0, 0.05 mol/L of Tris-HCl). The mixture was left in a shaker operating at 200 rpm for 10 to 240 min to reach equilibrium. After reaching adsorption equilibrium, the supernatant and the solid were separated by using a permanent magnet. BSA concentrations were measured by a UV-2501PC spectrophotometer at 595 nm. The amounts of BSA adsorbed on the magnetic adsorbents were calculated from mass balance. The standard curve of BSA is *Y* = 0.867*X* + 0.033(*R*^2^ = 0.9975).

## Results and discussion

**Figure 1:**
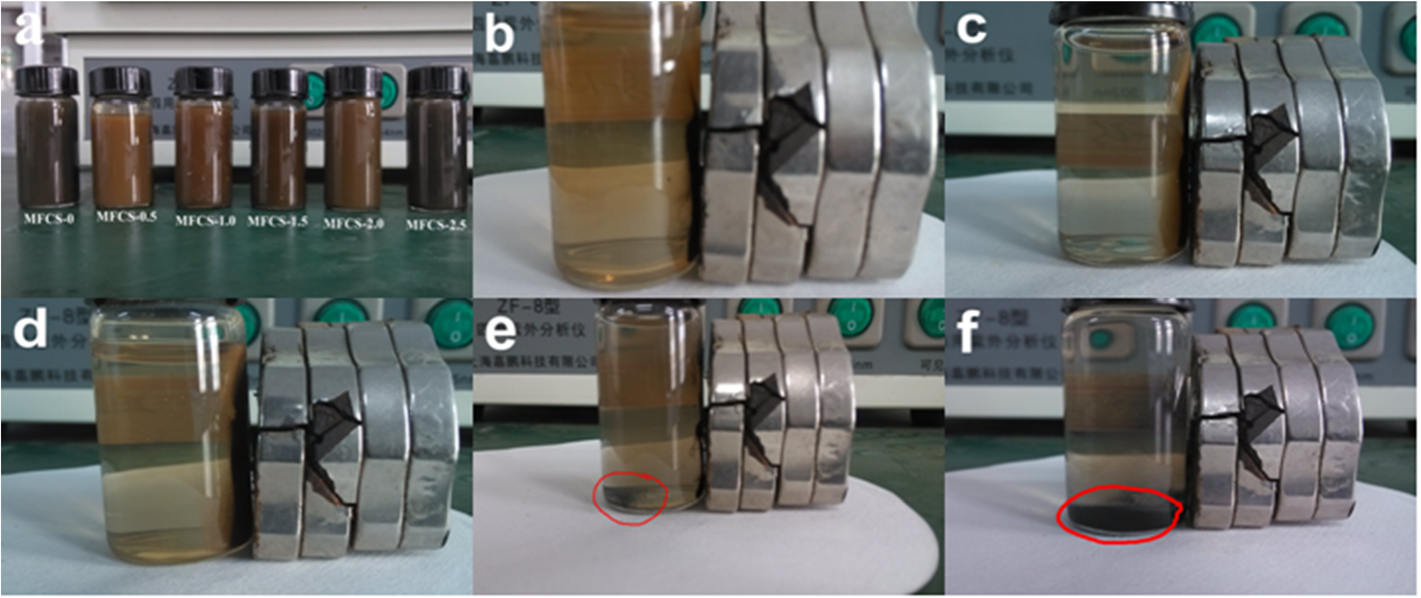
**Photos of the naked and CS-coated Fe**_**3**_**O**_**4 **_**NPs obtained. (a)** All MFCS. **(b)** MFCS-1/3. **(c)** MFCS-1/2. **(d)** MFCS-2/3. **(e)** MFCS-5/6. **(f)** MFCS-1.

All reactions rendered a black powder at the end of the process. However, a difference between the composite nanoparticles loaded with different amounts of chitosan was visually detected. Figure 1 presents photos of Fe_3_O_4_ coated with different amounts of chitosan. As shown in Figure 1a, the suspension color changed from black to tan and then turned to black with increasing amount of chitosan. Moreover, with increasing amount of chitosan of more than 1.25 g, there were lots of nonmagnetic black powder under the bottle (Figure 1e,f), which may be caused by the oxidization and aggregation of excessive chitosan.

**Figure 2:**
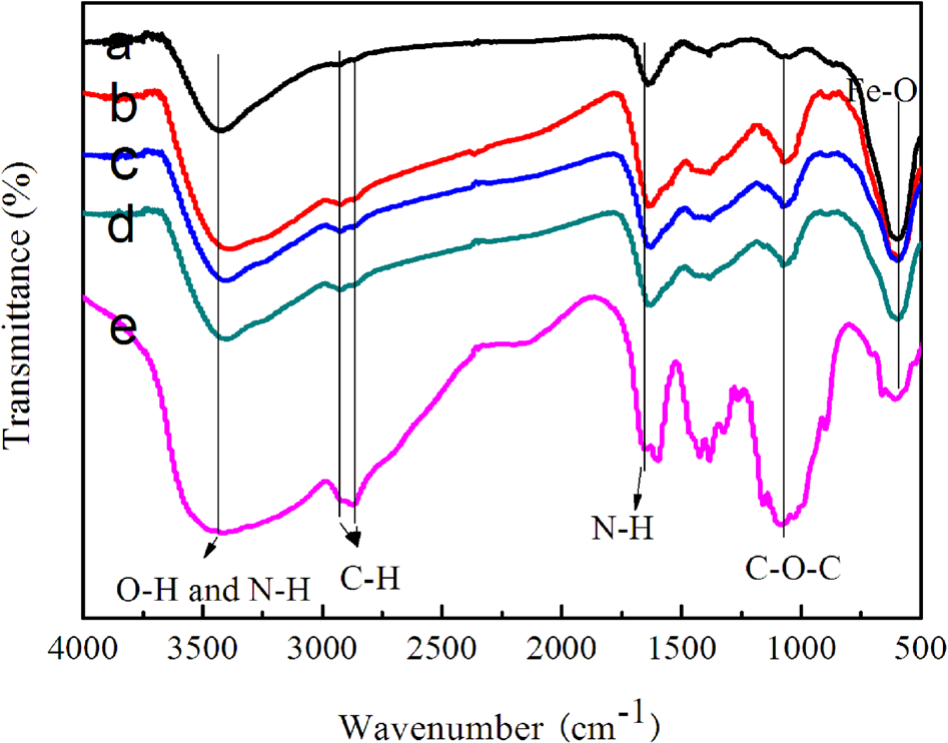
**FTIR spectra of the CS-coated Fe**_**3**_**O**_**4 **_**NPs obtained. (a)** MFCS-0. **(b)** MFCS-1/3. **(c)** MFCS-1/2. **(d)** MFCS-2/3. **(e)** Pure chitosan.

The functional groups of chitosan are very important for various applications, especially for biotechnological purposes. Therefore, the present functional groups should be kept even if the shape was changed into a new form; FTIR analyses were carried out. The FTIR spectra of MFCS-0, MFCS-1/3, MFCS-1/2, MFCS-2/3, and pure CS are given in Figure 2, which were exhaustively washed and magnetically recovered so that all the chitosan in the final products are chemically bound to the magnetic nanoparticles. In the spectrum of naked Fe_3_O_4_ (Figure 2a), the absorption at 586 cm^−1^ is assigned to the characteristic band of the Fe-O group [21]. For pure CS (Figure 2e), a broad band at 3,410 cm^−1^ assigned to the O-H stretching vibration can be seen, and the C-H group is manifested through peaks 2,922 and 2,861 cm^−1^. Of the characteristic absorption peaks of the primary amine (-NH_2_), one overlaps with the -OH band at 3,410 cm^−1^ and the second is visible at 1,654 cm^−1^. The band around 1,070 cm^−1^ is the stretching vibration of the C-O bond which is weaker in the spectrum of the composite nanoparticles (Figure 2b,c,d), suggesting the existence of weak chemical bonding between the Fe in Fe_3_O_4_ and the -OH group in CS [22]. These characteristic absorption peaks for Fe_3_O_4_ and CS demonstrate that the composite nanoparticles contain both Fe_3_O_4_ and chitosan.

**Figure 3:**
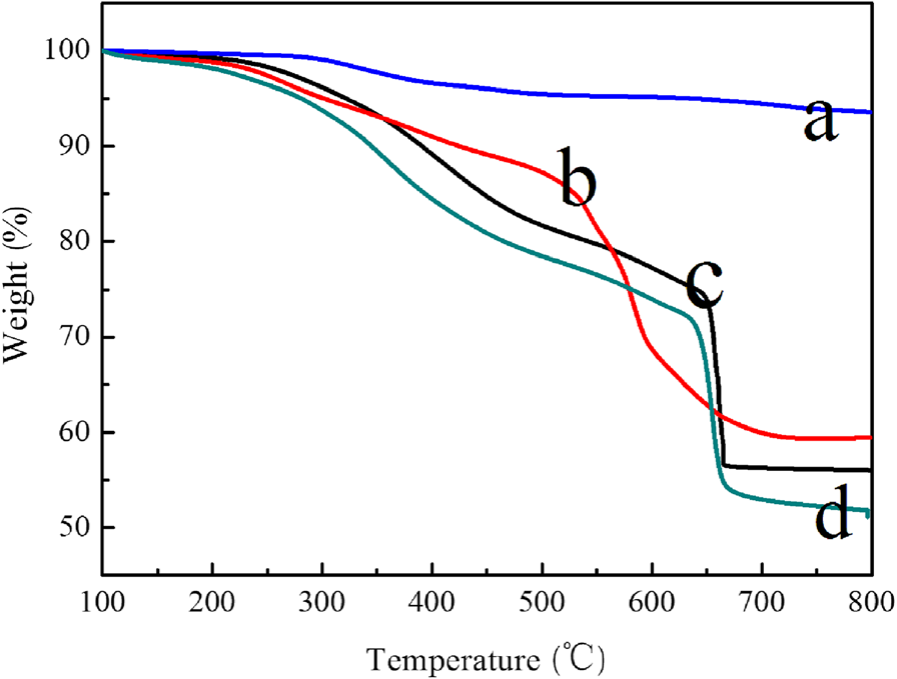
**TGA curves of the CS-coated Fe**_**3**_**O**_**4 **_**NPs obtained. (a)** MFCS-0. **(b)** MFCS-1/3. **(c)** MFCS-1/2. **(d)** MFCS-2/3.

The TGA curves of naked Fe_3_O_4_ and the magnetic composite nanoparticles are shown in Figure 3. For naked Fe_3_O_4_, the TGA curve showed that the weight loss over the temperature range 100°C to 800°C was about 6.4%. This might be due to the loss of the remaining water and agents. Compared with the TGA curves of the naked Fe_3_O_4_ NPs, those of the three kinds of CS-coated Fe_3_O_4_ NPs show that the decrease of the main mass of the as-synthesized NPs occurred from about 40% to 48%, attributed to the decomposition of CS anchored on the surface of the Fe_3_O_4_ NPs. It is thus demonstrated that considerable amounts of CS were successfully coated on the surface of the Fe_3_O_4_ NPs for further modification.

**Figure 4:**
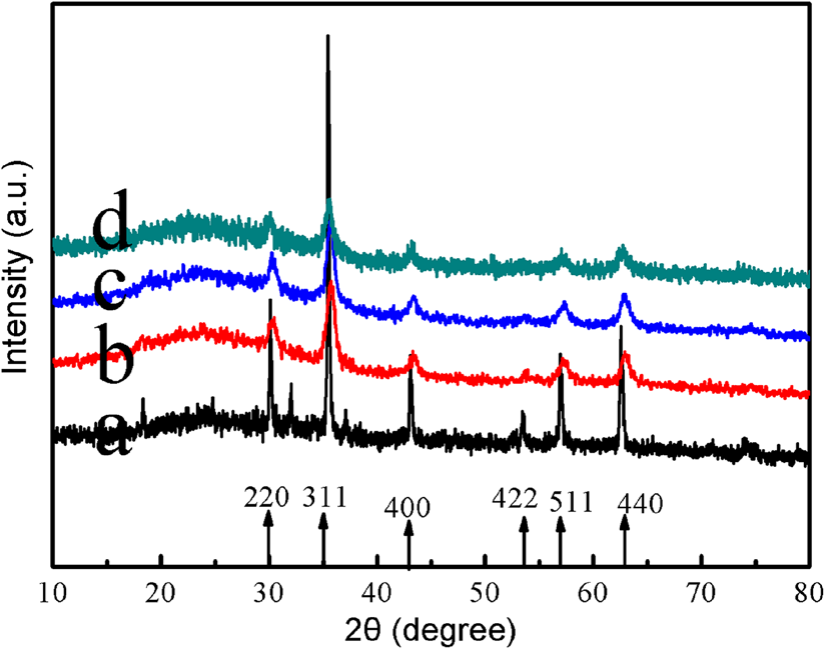
**The wide-angle XRD patterns of the CS-coated Fe**_**3**_**O**_**4 **_**NPs obtained. (a)** MFCS-0. **(b)** MFCS-1/3. **(c)** MFCS-1/2. **(d)** MFCS-2/3.

The crystal structures of the composite magnetic nanoparticles were characterized by X-ray diffraction in Figure 4. For the naked Fe_3_O_4_ NPs as prepared in this work, six characteristic peaks (2*θ* = 30.08°, 35.42°, 43.08°, 53.56°, 56.98°, and 62.62°) marked by their indices ((220), (311), (400), (422), (511), and (440)) were observed [23]. As shown in Figure 4b,c,d, these characteristic peaks can be seen in the composite magnetic nanoparticles, while the broad peak at 2*θ* = 17° to 27° was ascribed to chitosan, which indicated the existence of an amorphous structure [17].

**Figure 5:**
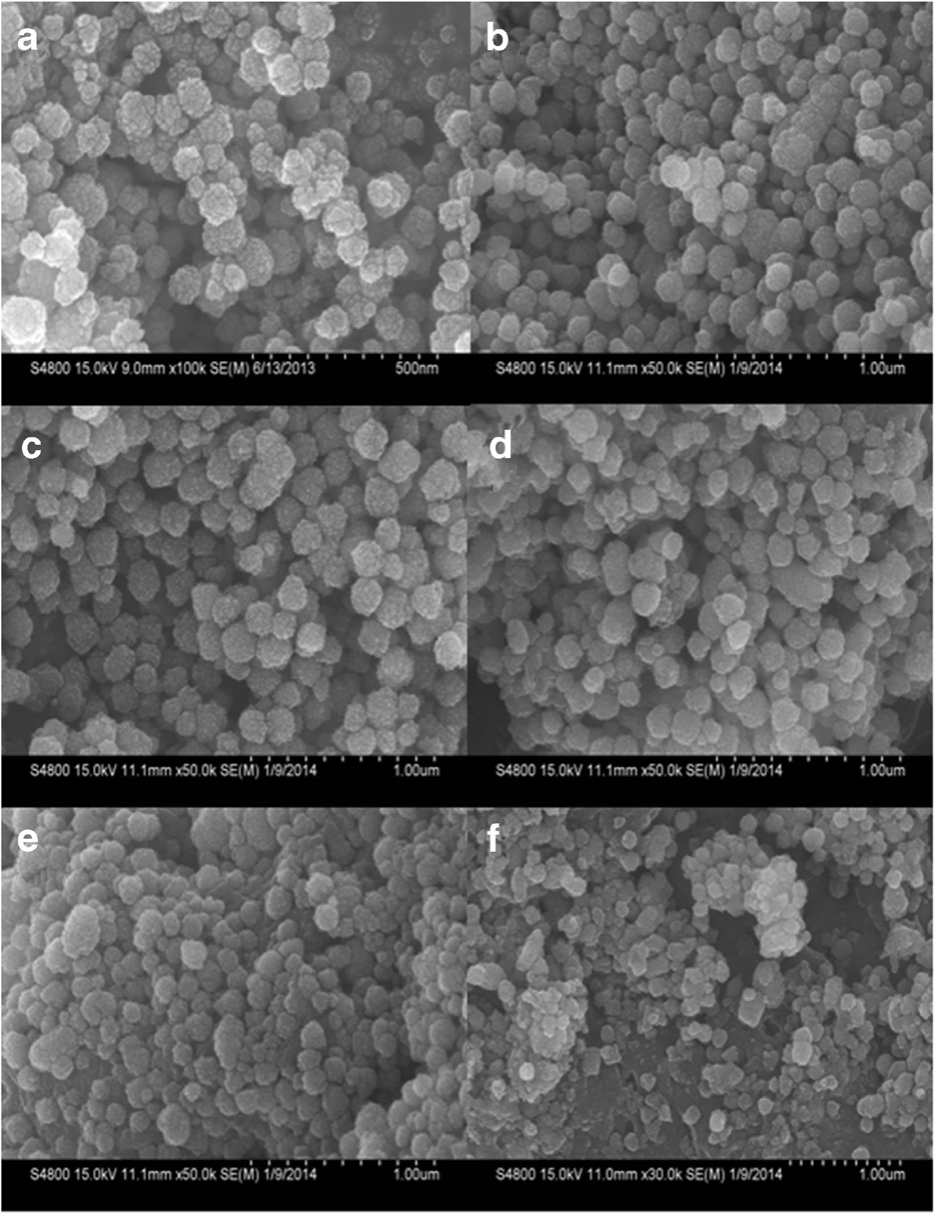
**SEM images of the CS-coated Fe**_**3**_**O**_**4 **_**NPs obtained. (a)** MFCS-0. **(b)** MFCS-1/3. **(c)** MFCS-1/2. **(d)** MFCS-2/3. **(e)** MFCS-5/6. **(f)** MFCS-1.

**Figure 6:**
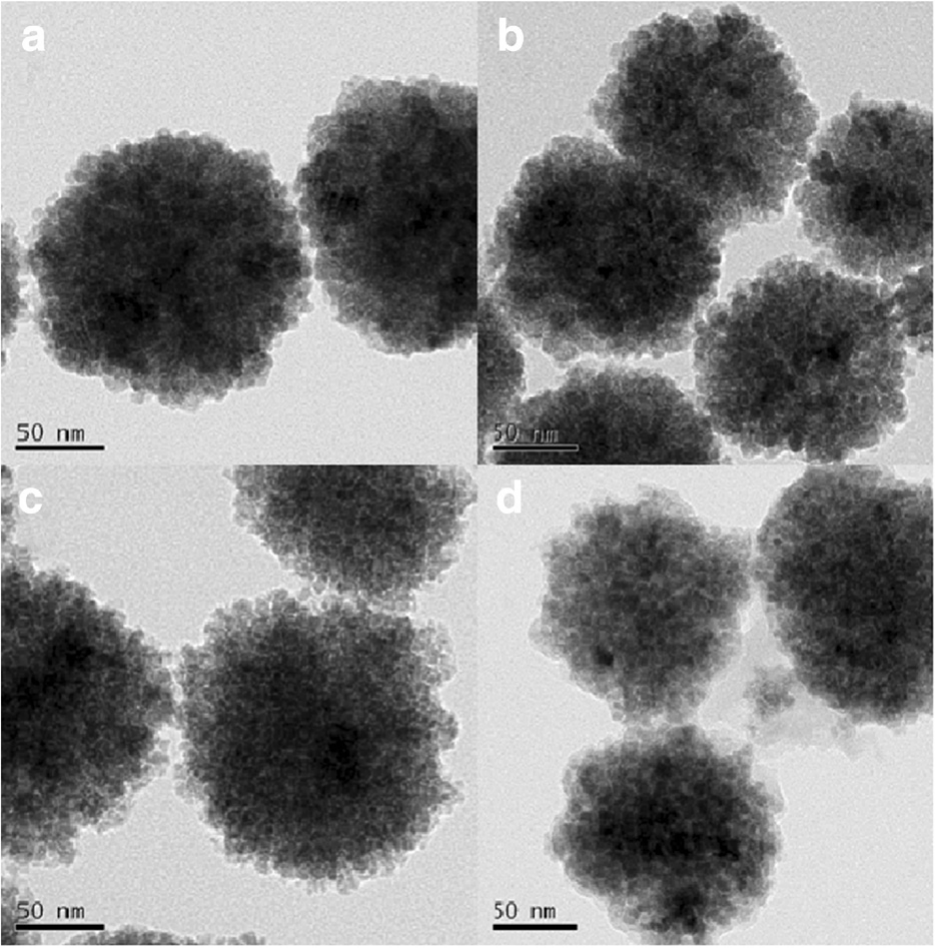
**TEM images of the CS-coated Fe**_**3**_**O**_**4 **_**NPs obtained. (a)** MFCS-0. **(b)** MFCS-1/3. **(c)** MFCS-1/2. **(d)** MFCS-2/3.

As seen in Figure 5, the surfaces of the spheres appear rough and composed of many small nanoparticles. However, the spheres tend to be uniform, and the surface of the nanoparticles became smoother with increasing weight ratios of chitosan/Fe from 0 to 1/2 (Figure 5a,b,c). When the weight ratio of chitosan/Fe was from 2/3 to 1, the CS-coated Fe_3_O_4_ NPs became morphologically rough and irregular and exhibited loss of structural cohesion (Figure 5d,e,f). In Figure 6, the spheres became smaller with increasing weight ratios of chitosan/Fe from 0 to 2/3.

**Figure 7:**
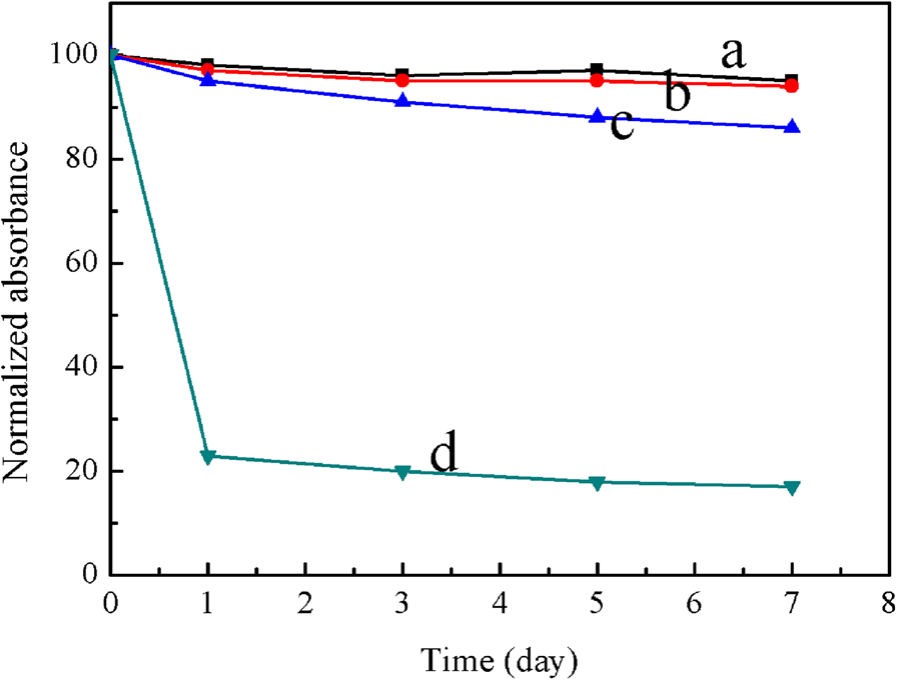
**Normalized UV-Vis absorbance of CS-coated Fe**_**3**_**O**_**4 **_**NPs.** In **(a)** water, **(b)** PBS plus 10% (*v*/*v*) fetal bovine serum, **(c)** PBS, and **(d)** NaCl (1.0 mol/L).

The stability of the CS-coated Fe_3_O_4_ NPs in this work was studied. We chose representative water, phosphate-buffered saline (PBS) plus 10% (*v*/*v*) fetal bovine serum, PBS, and NaCl (1.0 mol/L) as media in which CS-coated Fe_3_O_4_ NPs were dispersed to systematically investigate their stability by UV-visible absorbance spectroscopy at a fixed wavelength (450 nm). If nanoparticles are not stable and sedimentate rapidly, they can be monitored by a decreased absorbance as a function of time. Figure 7 shows that the CS-coated Fe_3_O_4_ NPs dispersed in water, PBS, and PBS plus 10% (*v*/*v*) fetal bovine serum present excellent stability, whereas those dispersed in high concentration of NaCl exhibit poor stability. These results suggest that the CS-coated Fe_3_O_4_ NPs dispersed in high concentration of NaCl aggregate rapidly, which is confirmed by the DLS result, as seen in Table 1.

**Table 1:** **Average hydrodynamic sizes of CS-coated Fe**_**3**_**O**_**4 **_**NPs dispersed in different media**

**Medium**	**Time**				
	**0 day**	**1 day**	**3 days**	**5 days**	**7 days**
Water	208.7 ± 12.6	214.2 ± 10.1	217.7 ± 9.5	224.4 ± 10.6	227.8 ± 13.4
PBS plus 10% (*v*/*v*) FBS	254.5 ± 5.7	260.1 ± 4.5	279.6 ± 7.7	288.9 ± 10.2	302.5 ± 9.8
PBS	286.6 ± 18.5	310.6 ± 35.8	347.0 ± 37.4	369.6 ± 41.2	404.4 ± 25.9
1.0 mol/L NaCl	542.7 ± 50.4	784.1 ± 45.7	1,009.2 ± 66.3	1,445.4 ± 57.1	1,667.8 ± 87.0

**Figure 8:**
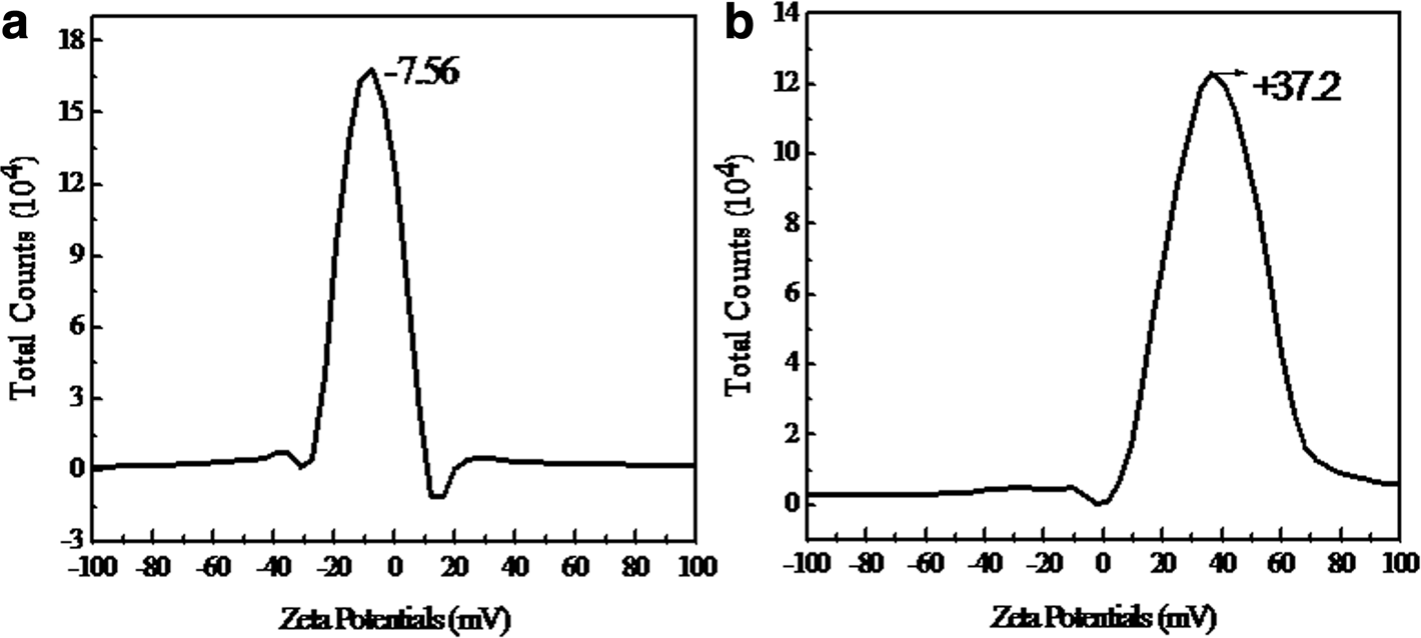
**The zeta potential of the as-prepared samples. (a)** MFCS-0. **(b)** MFCS-1/2.

The electrostatic interaction of the magnetic nanoparticles can be controlled by variation in their surface charges, which can be determined by measuring the zeta potential of these particles. Compared with that of naked Fe_3_O_4_ NPs (Figure 8a), the zeta potential of MFCS-1/2 possessed a higher positive charge (Figure 8b). This may be caused by the hydrogen of the amino group (-NH_2_) in chitosan. Thus, this indicated that the modification with CS on Fe_3_O_4_ NPs was successful.

**Figure 9:**
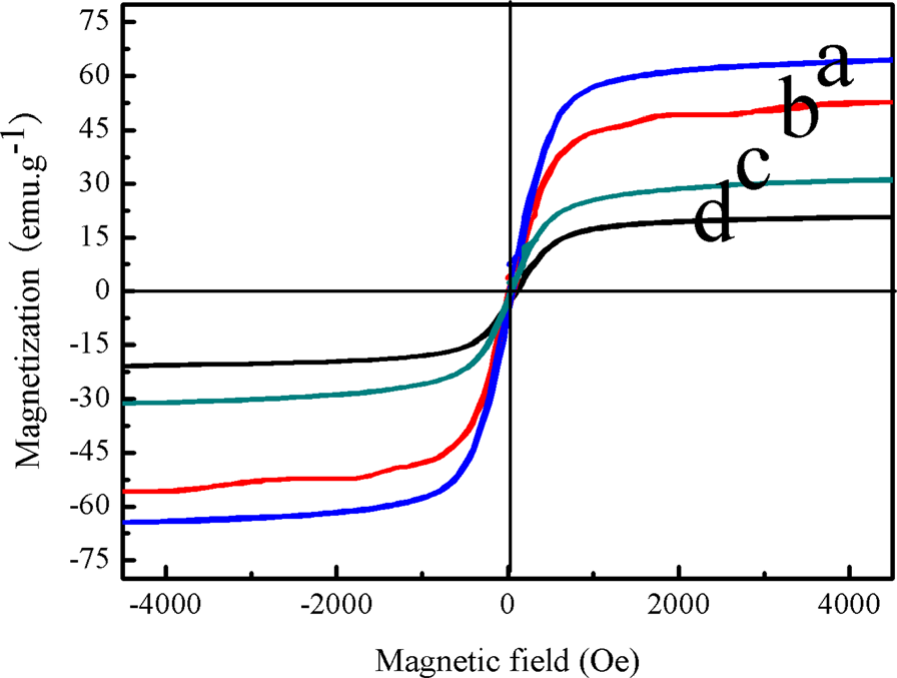
**Magnetization curves measured for the CS-coated Fe**_**3**_**O**_**4 **_**NPs obtained. (a)** MFCS-0. **(b)** MFCS-1/3. **(c)** MFCS-1/2. **(d)** MFCS-2/3.

The magnetic properties of the as-synthesized NPs after being coated with CS are a prerequisite for magnetic guiding application. To gain a better understanding of the magnetic properties of the as-synthesized NPs, the magnetization curves of different amounts of CS coated on the surface of the Fe_3_O_4_ NPs were measured. As shown in Figure 9, the saturation magnetization values of the CS-coated Fe_3_O_4_ NPs synthesized with chitosan: MFCS-0, MFCS-1/3, MFCS-1/2, and MFCS-2/3, were 64.2, 52.5, 30.8, and 20.5 emu g^−1^, respectively. This trend can likely be attributed to the higher weight fraction of chitosan.

**Figure 10:**
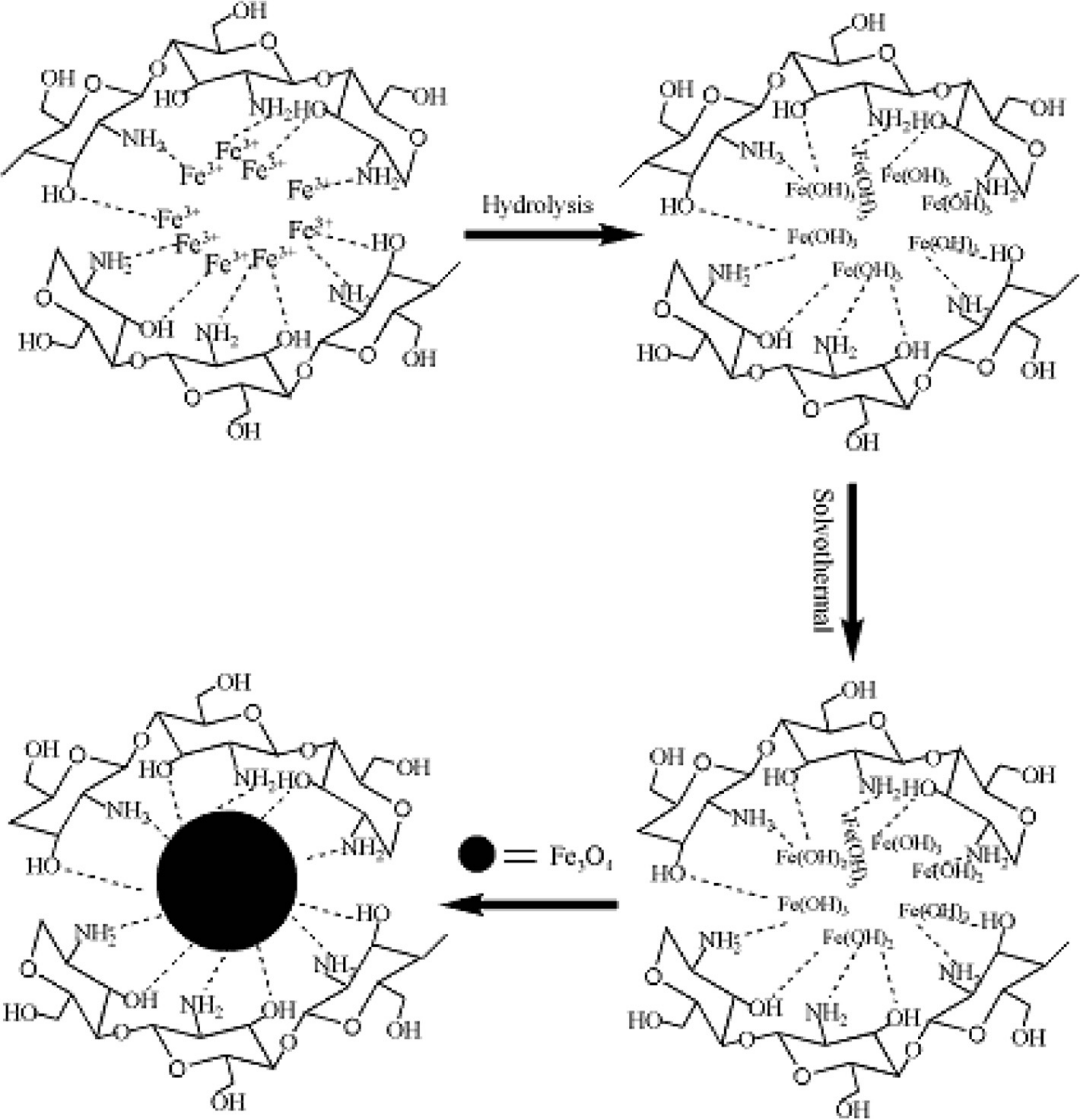
**A schematic showing the formation mechanism of the CS-coated Fe**_**3**_**O**_**4 **_**NPs by the solvothermal method.**

In the experiment, Fe(OH)_3_ was formed through the hydrolysis of FeCl_3_ · 6H_2_O, then Fe(OH)_2_ was obtained through the reduction of Fe(OH)_3_ with ethylene glycol at high temperature, and finally Fe(OH)_3_ and the newly produced Fe(OH)_2_ formed a more stable Fe_3_O_4_ phase. As reported by Burke [24], Fe(III) ions are attached to the chitosan's surface by forming a complex compound in which Fe(III) ions act as the metal center while the ligands are the amine and -OH of chitosan. Therefore, the possible reaction describing the formation mechanism of the CS-coated Fe_3_O_4_ NPs can be expressed by Figure 10.

**Figure 11:**
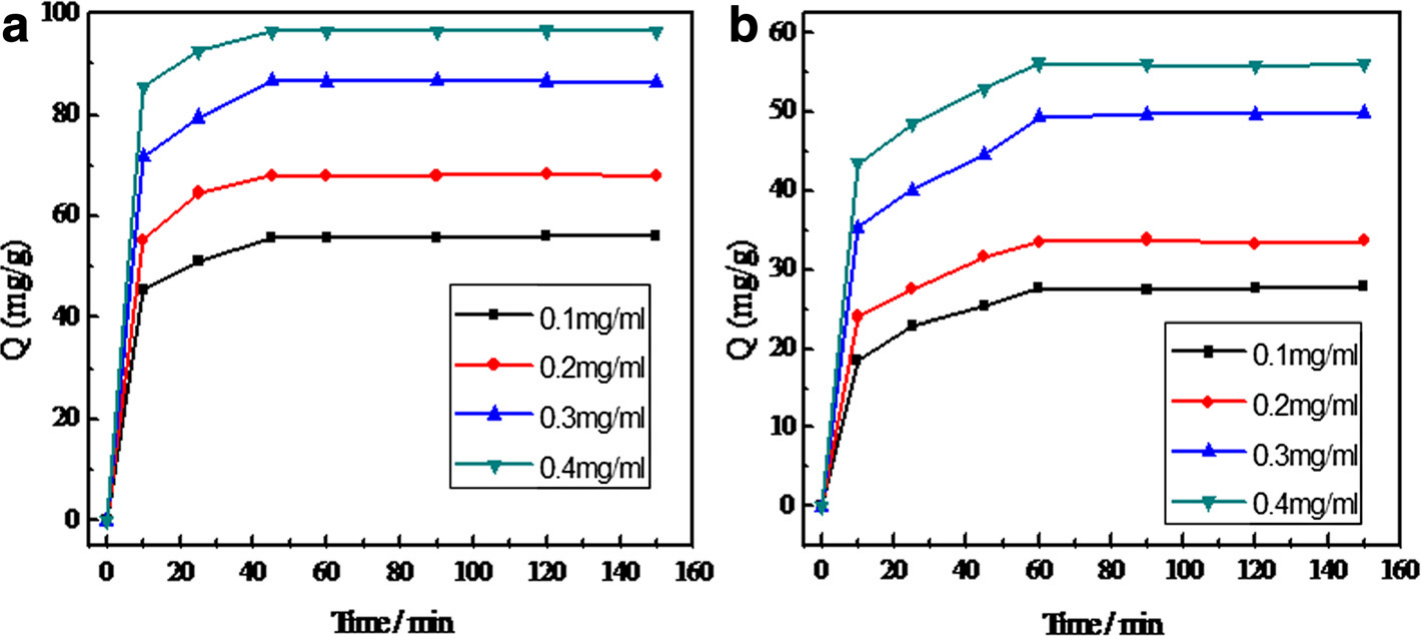
**Adsorption quantity of BSA with initial concentrations ranging from 100 to 400 mg/L. (a)** CS-coated Fe_3_O_4_ NPs. **(b)** Naked Fe_3_O_4_ NPs.

In order to investigate the adsorption capabilities and adsorption rate of the CS-coated Fe_3_O_4_ NPs, 10 mg of dried CS-coated Fe_3_O_4_ NPs were added into a 10.0-mL BSA aqueous solution. As illustrated in Figure 11a, the amount of adsorbed BSA increased with elapsed immersion time. Compared with naked Fe_3_O_4_ nanoparticles (Figure 11a), the CS-coated Fe_3_O_4_ NPs showed a higher BSA adsorption capacity (96.5 mg/g) and a fast adsorption rate (45 min) in aqueous solutions. This is due to the higher initial BSA concentration that provides a higher driving force for the molecules from the solution to the amide-functionalized CS-coated Fe_3_O_4_ NPs [25], resulting in more collisions between BSA molecules and active sites on the CS-coated Fe_3_O_4_ composites.

## Conclusions

In summary, a facile one-step solvothermal method was developed to prepare CS-coated Fe_3_O_4_ NPs with tunable magnetism, sizes, suspension stability, and surface charge. The size of the nanoparticles was about 150 nm, and chitosan made up 40% to 48.0% of the weight of the modified Fe_3_O_4_ NPs. Compared with Fe_3_O_4_ nanoparticles, the CS-coated Fe_3_O_4_ NPs showed a higher BSA adsorption capacity. This work revealed a promising method for the recovery of slaughtered animal blood by using magnetic separation technology.

## Competing interests

The authors declare that they have no competing interests.

## Authors’ contributions

MS carried out the total experiment and wrote the manuscript. WPJ participated in the data analysis. GDF supervised the project. GC, YMJ, and YJY provided the facilities and discussions related to them. WYT participated in the detection of the VSM and TEM. All authors read and approved the final manuscript.
